# Post-traumatic glenohumeral cartilage lesions: a systematic review

**DOI:** 10.1186/1471-2474-9-107

**Published:** 2008-07-23

**Authors:** Heidi Ruckstuhl, Eling D de Bruin, Edgar Stussi, Benedicte Vanwanseele

**Affiliations:** 1 Department of Mechanical and Process Engineering, Institute for Biomechanics, ETH Zurich, 8093 Zurich, Switzerland; 2 Institute of Human Movement Sciences and Sport, ETH Zurich, 8092 Zurich, Switzerland; 3 Department of Rheumatology and Institute of Physical Medicine, University Hospital Zurich, 8091 Zurich, Switzerland; 4 University of Sydney, School of Exercise and Sport Science, PO Box 170, Lidcombe NSW 1825, Australia

## Abstract

**Background:**

Any cartilage damage to the glenohumeral joint should be avoided, as these damages may result in osteoarthritis of the shoulder. To understand the pathomechanism leading to shoulder cartilage damage, we conducted a systematic review on the subject of articular cartilage lesions caused by traumas where non impression fracture of the subchondral bone is present.

**Methods:**

PubMed (MEDLINE), ScienceDirect (EMBASE, BIOBASE, BIOSIS Previews) and the COCHRANE database of systematic reviews were systematically scanned using a defined search strategy to identify relevant articles in this field of research. First selection was done based on abstracts according to specific criteria, where the methodological quality in selected full text articles was assessed by two reviewers. Agreement between raters was investigated using percentage agreement and Cohen's Kappa statistic. The traumatic events were divided into two categories: 1) acute trauma which refers to any single impact situation which directly damages the articular cartilage, and 2) chronic trauma which means cartilage lesions due to overuse or disuse of the shoulder joint.

**Results:**

The agreement on data quality between the two reviewers was 93% with a Kappa value of 0.79 indicating an agreement considered to be 'substantial'. It was found that acute trauma on the shoulder causes humeral articular cartilage to disrupt from the underlying bone. The pathomechanism is said to be due to compression or shearing, which can be caused by a sudden subluxation or dislocation. However, such impact lesions are rarely reported. In the case of chronic trauma glenohumeral cartilage degeneration is a result of overuse and is associated to other shoulder joint pathologies. In these latter cases it is the rotator cuff which is injured first. This can result in instability and consequent impingement which may progress to glenohumeral cartilage damage.

**Conclusion:**

The great majority of glenohumeral cartilage lesions without any bony lesions are the results of overuse. Glenohumeral cartilage lesions with an intact subchondral bone and caused by an acute trauma are either rare or overlooked. And at increased risk for such cartilage lesions are active sportsmen with high shoulder demand or athletes prone to shoulder injury.

## Background

Any cartilage damage to the glenohumeral (GH) joint should be avoided, as these damages will result in osteoarthritis (OA) of the shoulder [1,2], also known as omarthrosis. Full-thickness articular cartilage defects have limited capacity to heal and a slight incongruity caused by articular cartilage damage generates overload situations [3,4]. Therefore, early detection of structural changes (thinning, lesions) to the hyaline cartilage is necessary [4,5]. Etiology of articular cartilage abnormalities in the GH joint is quite diverse and includes avascular necrosis, chondrolysis, idiopathic focal defects, osteoarthritis, osteochondritis dissecans, postsurgical cartilage abnormalities (iatrogenic injuries), and post-traumatic defects [4,6]. For this review, only cartilage damages due to traumatic events are considered.

Traumatic events can be grouped into two categories [7]: acute and chronic trauma. Acute trauma refers to any single impact situation which directly damages the articular cartilage, whereas chronic trauma means cartilage lesions due to overuse or disuse of the GH joint. Chronic traumas represent only indirect damage to GH cartilage. It is well known that highly stressed shoulders (e.g. wheelchair users [8], throwing athletes [9]) are prone to develop structural abnormalities. As a consequence of such an injury, the mechanics of the glenohumeral motion may be altered, which in turn can lead to overload situations and cartilage damage. For example, tearing of rotator cuff (RC) and GH-OA are two common pathologies [1,10-12].

A clinical problem with regard to traumatic events is the early detection of changes in the cartilage structure without concomitant bony fracture, e.g. when GH cartilage lesions do not present together with subchondral fracture in patients with traumatic anterior instability. Because cartilage damage may be subtle, such lesions may remain undetected which could have substantial impact on the level of independent functioning in daily life. Therefore, it is desirable to detect such cartilage changes in clinical settings. In a first step, however, it is necessary to get a clear picture about GH articular cartilage lesions where the subchondral bone is not fractured.

The main aim of this study is to review the incidence of post-traumatic GH articular cartilage degeneration/lesions with non-fractured subchondral bone and to group them according to their traumatic event. Further research questions are: (i) Is there a specific population group predisposed to traumatic shoulder cartilage lesions? (ii) What is a typical pathomechanism leading to GH cartilage damage? (iii) What is the level of evidence of publications on the topic of GH cartilage damage?

## Methods

A computer-aided search of PubMed (MEDLINE), ScienceDirect (EMBASE, BIOBASE, BIOSIS Previews) and the COCHRANE database of systematic reviews was performed at August 2007 to identify relevant articles in this field of research. Additionally, the reference lists of the identified studies were checked for other suitable studies. The search strategy in PubMed included a combination of the following search terms: "humans" AND "shoulder" AND "cartilage, articular". To identify relevant articles through ScienceDirect and Cochrane databases, the two search terms "shoulder" AND "cartilage" were used. Restrictions were made regarding the language of the publication. Only papers in English or German were included into the review process.

One reviewer (HR) assessed the title and abstract of each identified study. Abstracts were included in the full-text review when their abstracts satisfied following criteria:

▪ Only studies debating glenohumeral cartilage lesions and degeneration are included. Joint space narrowing as an outcome is not of interest, as this radiological measure is only an indirect assessment of cartilage degeneration and does not give any information about possible lesions of the cartilage.

▪ Studies had to discuss the effects of traumatic events on glenohumeral cartilage degeneration. Cartilage lesions due to avascular necrosis, chondrolysis, idiopathic focal defects, osteoarthritis, osteochondritis dissecans, and postsurgical cartilage abnormalities are not included. Traumatic events are divided into two categories: (1) acute traumas refer to any impact situation which directly damages the articular cartilage and (2) chronic traumas refer to any articular cartilage damage in conjugation with other pathologies of the shoulder joint, therefore referred to as overuse injuries. Three types of chronic traumas are distinguished. Either the cartilage damage is associated with (i) RC injury, (ii) instability, or (iii) impingement syndrome. This classification is in accordance with results of Jobe and Jobe [13], who grouped athletic injuries of the GH joint into the mentioned three categories.

▪ For this study, only articular cartilage lesions are considered, whereas the subchondral bone is not fractured, i.e. there is no impression fracture. Therefore, studies about fractures to the humeral head (e.g. cuff-tear arthropathy [11], Hill-Sachs lesion [14,15]) or the glenoid cavity which indirectly affect the cartilage are excluded. Also glenolabral articular disruption (GLAD) lesions [16,17], where anteroinferior glenoidal cartilage damage is a consequence of an anteroinferior labral tear and not of a direct trauma, are not included in the review process. Animal studies are not included as well.

Two reviewers (HR and EDdB) independently assessed the study quality of the included reports according to four selected criteria of the checklist of Downs and Black [18]. In the majority of the reviewed articles the occurrence of cartilage lesions was discussed retrospectively and the effect of health care intervention on clinical outcome was rarely described. Therefore only items related to the quality of reporting are used: description of hypothesis/aim/objective (item 1), clear description of the method (item 2), characteristics of the patients (item 3), and detailed description of the main results (item 6). For each criterion, two rating categories were chosen: yes (match criteria), no (does not match). A maximum score of 4 points could be obtained. Percentage agreement and Cohen's Kappa statistic [19] were calculated with GRAPHPAD Software (Version 2002) and were interpreted in accordance with Landis and Koch's benchmarks for assessing the agreement between raters [20]: poor (<0), slight (.0–.20), fair (.21–.40), moderate (.41–.60), substantial (.61–.80), and almost perfect (.81–1.0).

In addition to assessing the quality of the study, the level of evidence of each of the reviewed studies was determined according to the classification of Dick [21].

## Results

### Selection of articles and methodological quality

**Figure 1:**
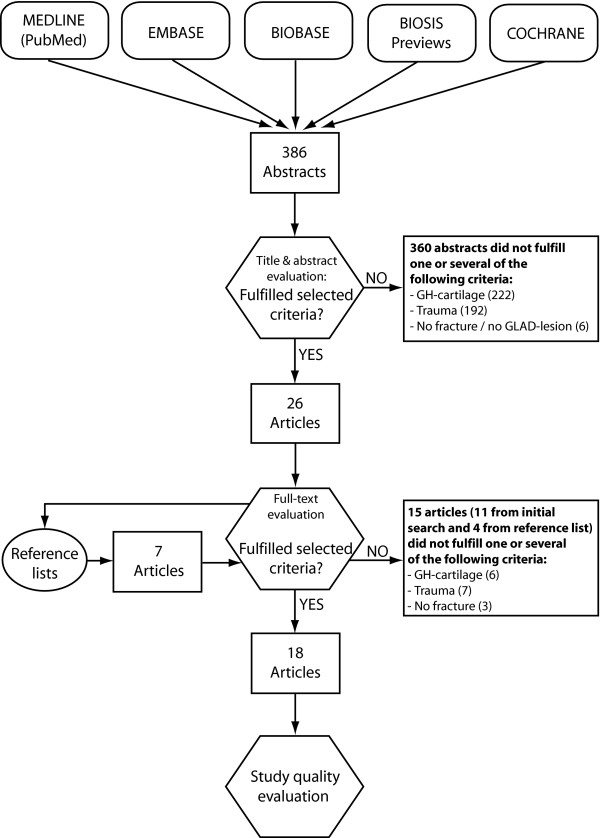
**Databases and selection process**. Procedure for selection of the studies with utilized databases for the literature search and the applied selection criteria.

The literature search yielded 386 abstracts for consideration. After abstract evaluation and application of the inclusion criteria, 26 articles entered the full-text review. Checking of the reference list identified another 7 papers. After full-text evaluation of the overall 33 articles and further application of the inclusion criteria, 18 papers were included in the review process [1,4,12,22-36] (Figure 1). Thereof, two papers discussed the influence of acute traumatic events on humeral cartilage degeneration [23,28]. Fifteen papers reported the incidence of GH cartilage degeneration due to chronic inappropriate mechanical stresses [1,4,12,22,24-27,29-33,35,36]. In one paper, acute as well as chronic traumas were described [34]. The quality scores of studies ranged from 2 to 4 points out of a maximum of 4 points. The quality score (mean and s.d.) was 3.2 ± 0.8 points. The agreement on data quality between the two reviewers was 93%. The Kappa value was 0.79 with a confidence interval of 0.59–0.99 indicating an agreement considered to be 'substantial'. Each of the reviewed studies was classified as having level IV evidence.

### Acute traumas

**Figure 2:**
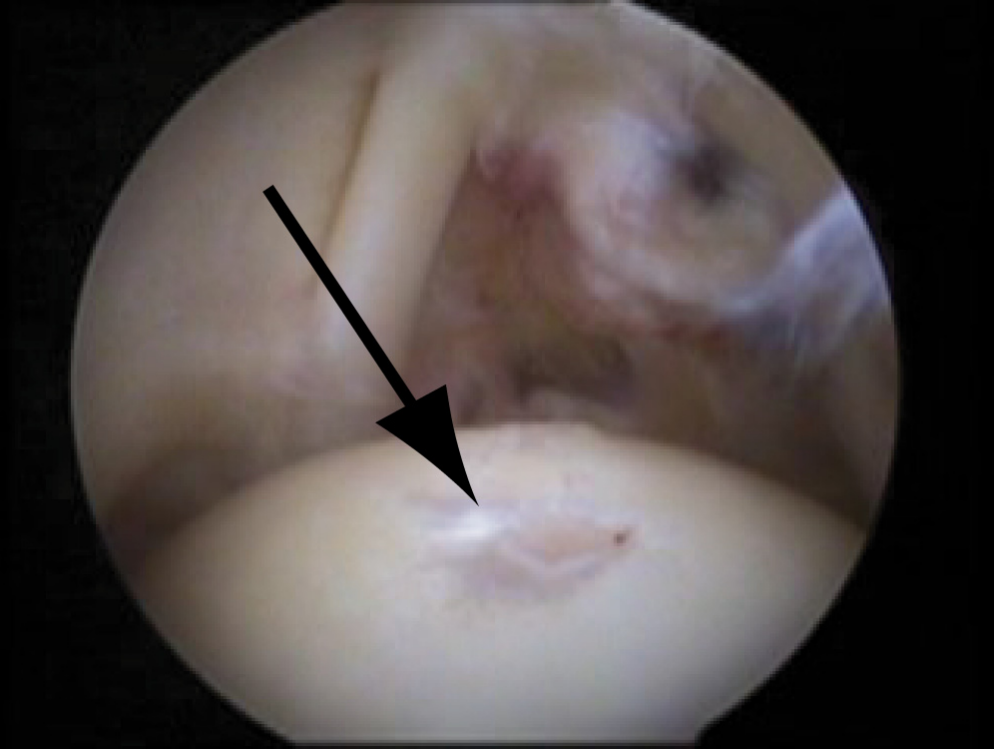
**Humeral cartilage defect**. Arthroscopic view of the humeral head with a focal cartilage defect (arrow), provided by MD Jacek Krzycki, Swiss Paraplegic Center Nottwil, Switzerland.

Shoulder cartilage lesions after a single trauma are rarely reported. In such cases however, a high impact may cause the articular cartilage to get disrupted, whereas in many cases there is destruction of the cartilage down to the bone. Thereby, the subchondral bone might be slightly affected as well (bone bruising [28] or abnormal marrow [23]). Articular cartilage lesions of the humeral head [23,28,34] are observed with a lesions size up to 6 cm^2^. An example of a focal humeral cartilage lesion is shown in Figure 2. Cartilage lesions on the convex surface of the humeral head may be due to high compressive or shearing forces [23,28]. Dislocation and subluxation are often reported as possible causes for these high forces. Defects are reported posterosuperior, inferior or central. The location of the humeral head defect is dependent on the mechanism of injury, that is, dependent on the manner of the impact. Physical active persons are at risk, as any injury puts the shoulder joint under high stresses (e.g. fall on shoulder [28], crash with another player [23]). Especially athletes in contact sports may be prone to this type of injury. Please refer to [Additional file 1] for a summary of the papers related to acute traumas.

### Chronic traumas

The incidence of glenohumeral cartilage lesions in subjects with full-thickness RC tears is reported to be between 7% and 28% [26,31], with lesions up to 6 cm^2^. In patients with partial- or full-thickness tears of the RC, degenerative changes of the humeral head [25] and the glenoidal cavity [25,30] are more common compared to subjects without tears. Furthermore, the defect size of the cartilage damage is larger for both humerus and glenoid if RC tears are present [1]. The defects are mainly located at the posterosuperior part of the humeral head [1,25] (similar to the Hill-Sachs lesion) and inferior part of the glenoid [1,25,30]. No differences in the location of cartilage degeneration between the RC and non-RC tear group are observed [25]. In the presence of cartilage lesions of the humerus [12,25] or glenoid [12,25], it is more likely to observe associated RC degeneration or tears. The majority (43% to 76%) of the patients with cartilage lesions have associated RC degeneration or tears, whereas in subjects without any cartilage degeneration only 0% to 19% experience RC degeneration or tears. Kissing lesions of the GH joint are observed in the following three positions: (i) 90° of flexion, (ii) 90° of abduction with external rotation, and (iii) maximal elevation [1]. In the cadaveric studies, the authors excluded the shoulders in case of a known rheumatoid disease or upper limb trauma [25], in case of a history of systemic disease [1], and in case of glenoid deformity caused by glenoid fractures or bone tumour [30]. In the cadaveric study done by Petersson [12], only 3 out of 76 subjects (4%) had known shoulder distress during life-time. Please refer to [Additional file 2] for a summary of the papers related to RC injury.

The incidence of humeral and glenoidal cartilage lesions in unstable shoulders is reported to be 25% [22] and 57% [35], respectively. Anterior instability is observed most frequently [22,35]. Chondral damages are more common in unstable shoulders when time from injury to surgery is higher [22]. The humeral defect has a size up to 5 cm^2^, whereas the reported glenoidal defects are smaller. There is no specific defect location on the humerus or glenoid and no associated cartilage degeneration and direction of instability [22]. In patients with instability the associated pathology is mainly labral damage [4,33,35,36]. A history of dislocation exists in many cases of instability [4,35,36]. Athletes at high risk for shoulder injuries may be predisposed to dislocate the shoulder, e.g. a contact game player [4,36]. Please refer to [Additional file 3] for a summary of the papers related to instability.

The incidence for humeral and glenoidal cartilage lesions in patients with impingement diagnosis is reported at an average value of 16% and 10%, respectively [24,27,29,32]. In the study of Ellman et al. [24] only cases with clinically diagnosed impingement who had full-thickness cartilage loss at the time of arthroscopy are reported. This group represents about 6% of the patients who had arthroscopy for impingement during the time of the study [6]. In summary it can be stated that mainly persons with high shoulder demand during their activity (e.g. weightlifters, throwing athletes) are diagnosed with impingement [24,29,32]. Seventeen percent of professional overhand throwing athletes with pain during the late cocking or acceleration phase have a humeral defect located close to the insertion of the supraspinatus (SSP) [32]. In this subgroup, associated RC fraying is seen in 71%. Please refer to [Additional file 4] for a summary of the papers related to impingement syndrome.

**Figure 3:**
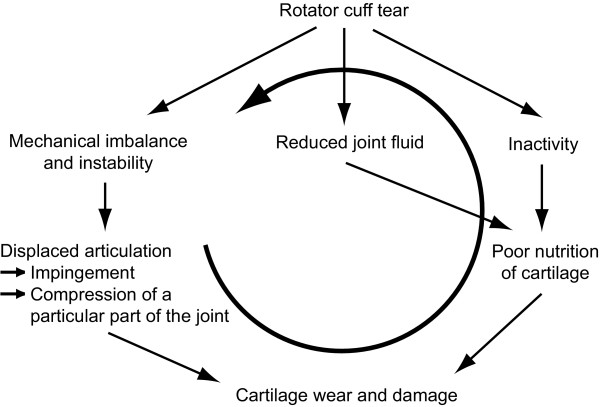
**Pathomechanism leading to chronic post-traumatic GH cartilage defects**. In case of chronic post-traumatic GH cartilage defects, the RC is injured first. This may result to instability and following impingement and to a malnourished cartilage. In addition, RC tears might be further promoted. In the final stages, this mechanism might lead to GH articular cartilage damage.

Several similar pathomechanism of chronic post-traumatic GH cartilage defects are suggested. In summary, it can be stated that RC tears can lead to mechanical imbalance [12], instability [1,4], and inactivity [11] in addition to the reduced joint fluid because the joint space is not closed any more [11,12]. These factors all predispose the articular cartilage to degenerate. Due to mechanical imbalance and instability, a displaced articulation compresses a particular part of the joint surface [1] and increases wear of cartilage [12]. Furthermore, a reduced synovial fluid quantity and inactivity might result in malnourished cartilage [11,12]. The described pathomechanism leading to chronic post-traumatic GH cartilage defects is illustrated in Figure 3.

## Discussion

We found only few reports on post-traumatic GH cartilage lesions without any bony lesions which lets assume that this type of shoulder pathology is not prevalent. This fact is supported by the finding that only 3% of all reported cases of osteoarthritis concerns the shoulder joint [37] and that the incidence rate of GH cartilage lesions at arthroscopy is approximately 5% [6]. Carroll et al. [23] searched their computer database for MR imaging of the shoulder and found superior humeral cartilage defects in less than 1% of patients. However, it is suggested that shoulder cartilage lesions may be overlooked on MR imaging [23]. This could be especially true for individuals with highly loaded shoulders (e.g. wheelchair users [8], throwing athletes [9]), where structural abnormalities are often reported. Therefore, appropriate instrumentation is needed to detect cartilage lesions and this should preferably be implemented in those cases where a suspicion of cartilage degeneration seems appropriate. Currently, the gold standard in vivo is arthroscopy (as also shown in the knee joint [38]), or analysis of shoulder joint cadavers in vitro. Miller and Savoie [31] state that direct visualization through the arthroscope provides the best opportunity for the detection of subtle, intra-articular abnormalities. The diagnostic effectiveness of MR arthrography to detect cartilage lesions is moderate [27]. Gartsman and Taverna [26] found no evidence of cartilage loss at preoperative plain radiographs and MRI studies – whereas cartilage thinning and loss could be observed with arthroscopy.

One should be aware that post-traumatic glenohumeral cartilage lesions are often present with subchondral bony lesions in patients with anterior instability [14,15], which we didn't include into the review. It might be speculated that symptoms in patients with shoulder cartilage lesions and an intact subchondral bone are less severe than in patients with osteochondral lesions, which might affect the frequency of seeking medical care.

In case of traumatic lesions, there are two main categories to distinguish. First, the cartilage is affected directly by an accident. This single impact (also described as acute trauma) on the shoulder causes humeral cartilage to disrupt from the underlying bone. In the second case (chronic trauma), glenohumeral cartilage degeneration is a result of chronic overuse and is associated to other shoulder joint pathologies such as RC injury, instability and impingement syndrome. For both types of trauma – acute and chronic – active sportsmen with high shoulder demand (e.g. throwers [9,32]) or athletes prone to shoulder injury (e.g. rugby player [28,39]) are at a higher risk for GH cartilage lesions.

The pathomechanism of acute traumatic humeral cartilage defects is said to be due to compression or shearing [23,28], which can be caused by a sudden subluxation or dislocation [6,28]. In case of chronic post-traumatic GH cartilage defects, the RC is injured first. This can result to instability and following impingement while RC tears are further promoted. In the final stages, this mechanism might lead to GH articular cartilage damage. It is important to notice that RC tears seem to be caused by progressive wear and impingement rather than to trauma [11], that RC tendons have regions of reduced blood supply [40] making them vulnerable to degenerate with age, and that the incidence of RC tears is low in persons younger than 40 years [25,41-43].

Limitations of this study are as follows: (i) we found only three reports describing pure humeral cartilage damage due to an acute trauma which limit conclusions about this type of injury. (ii) clear separation of RC injury, instability, and impingement syndrome might not be possible, as these factors may be also conjointly existent: shoulder muscles are powerful stabilizers [44], in case of impingement diagnosis, the RC is often injured as well [27,29] and impingement and instability are two entities which are often related [45]. Furthermore, different types of impingement are described: subacromial impingement syndrome (SSP tendinopathy or partial tear, narrowing of the subacromial space due to a subacromial spur, osteoarthritis of the AC joint, or subacromial bursitis [27]) and internal impingement (the undersurface of the RC contacts the posterosuperior glenoid in the abducted and externally rotated position [29,32]). (iii) In case of three cadaveric studies related to RC tears [1,12,30], the individual history of traumas is not known. (iv) As RC tears are more frequent with increasing age, degenerative changes of the articular cartilage in the elderly might be more frequent. That is, the age of the patients has to be considered when interpreting data. (v) A further limitation of this review is related to the level of evidence of the studies that we included in our review. The application of diagnostic methods into daily clinical practice should be based on scientific evidence. We were not able to identify any study that implemented an attempt to diagnose GH cartilage damage in an at risk population in a prospective, randomised study type with any type of diagnostic test. Most studies included in this review used historic, non-randomised cohort or case control study designs. This point reveals the importance of developing diagnostic tests that can be implemented in studies with a high level of evidence.

## Conclusion

The great majority of glenohumeral cartilage lesions without any bony lesions are the results of overuse secondary to chronic traumas associated with rotator cuff injury, instability, or impingement syndrome. Glenohumeral cartilage lesions with an intact subchondral bone and caused by an acute trauma are either rare or overlooked. And high quality research designs in "at risk populations" should be implemented in the future to determine the value of MRI, MR arthrography, radiography or alternative diagnostic tools in diagnosing GH cartilage degeneration.

## Competing interests

The authors declare that they have no competing interests.

## Authors' contributions

HR drafted the manuscript, did first selection of articles, and assessed the quality of the papers. EDdB gave important inputs for the methodic part of this paper, assessed the quality of the papers, performed the statistical analysis, and revised the manuscript critically for its content. ES revised the manuscript critically for its content. BV helped to draft and to correct the manuscript. All authors read and approved the final manuscript.

## Pre-publication history

The pre-publication history for this paper can be accessed here:



## Supplementary Material

Additional File 1 Acute traumas leading to GH cartilage lesions. Abbreviations: ABER, abducted and externally rotated; Age (mean, range); ant, anterior; AS, Arthroscopy; cent, central; Disloc., Dislocation; Deg., Degeneration; GH, glenohumeral; Glen., Glenoid; Hum., Humerus; Hyperlax., Hyperlaxity; Imping., Impingement; inf, inferior; Instab., Instability; Instr., Instrumentation; multidir., multidirectional; n, number of subjects; post, posterior; RC, rotator cuff; S, number of shoulders; sup, superior. Click here for file

Additional File 2 Chronic traumas leading to GH cartilage lesions due to RC injuries. ^1^Significant less than in the RC tear group; ^2^Significant less than 36% and 32%, respectively; ^3^Significant more common than in the non RC-tear group; ^4^Significant less than 76%. For abbreviations, see [Additional file 1]. Click here for file

Additional File 3 Chronic traumas leading to GH cartilage lesions due to instability. The first two articles discuss the incidence of GH degeneration in unstable shoulders. The remaining papers report on single cases where all patients show signs of humeral and/or glenoidal degeneration. ^1^Numerous dislocations; ^2^No history of dislocation but pain at throwing; ^3^Numerous subluxations post; ^4^Luxation ant-inf; ^5^Numerous dislocations ant. For abbreviations, see [Additional file 1]. Click here for file

Additional File 4 Chronic traumas leading to GH cartilage lesions due to impingement. ^1^Mainly sportive active men, e.g. weightlifter, tennis player; ^2^Near supraspinatus insertion. For abbreviations, see [Additional file 1]. Click here for file
