# Supplementary material for: Post-traumatic glenohumeral cartilage lesions: a systematic review
Source: BMC Musculoskelet Disord. 2008 Jul 23;9:107. doi: 10.1186/1471-2474-9-107 (PMC2503981; doi:10.1186/1471-2474-9-107)
Supplement: Additional File 1 — Acute traumas leading to GH cartilage lesions. Abbreviations: ABER, abducted and externally rotated; Age (mean, range); ant, anterior; AS, Arthroscopy; cent, central; Disloc., Dislocation; Deg., Degeneration; GH, glenohumeral; Glen., Glenoid; Hum., Humerus; Hyperlax., Hyperlaxity; Imping., Impingement; inf, inferior; Instab., Instability; Instr., Instrumentation; multidir., multidirectional; n, number of subjects; post, posterior; RC, rotator cuff; S, number of shoulders; sup, superior. [file 1471-2474-9-107-S1.doc]

| **Study** | **n** | **Age** | **Instr.** | **Lesion** | **Results** | **Defect detail** | **Activity** |
| --- | --- | --- | --- | --- | --- | --- | --- |
| Carroll et al. 2001 [23] | 7 | 34  (15-63) | AS / MRI | Focal articular cartilage lesion | Deg. Hum. | post-sup  up to 2×3 cm^2^ | - Contact sports - Weight lifting - Fast movements |
| Jeon and Wallace 2004 [28] | 1 | 20 | AS | Traumatic humeral articular cartilage shear lesion | Deg. Hum. | inf  70% of inf half | Contact sport (rugby) |
| Scheibel et al. 2004 [34] | 2 | 40  (23-56) | AS | Full-thickness articular cartilage defect | Deg. Hum. | cent  up to 1.6×1.6 cm^2^ |  |
