# Supplementary material for: Post-traumatic glenohumeral cartilage lesions: a systematic review
Source: BMC Musculoskelet Disord. 2008 Jul 23;9:107. doi: 10.1186/1471-2474-9-107 (PMC2503981; doi:10.1186/1471-2474-9-107)
Supplement: Additional File 2 — Chronic traumas leading to GH cartilage lesions due to RC injuries. 1Significant less than in the RC tear group; 2Significant less than 36% and 32%, respectively; 3Significant more common than in the non RC-tear group; 4Significant less than 76%. For abbreviations, see [Additional file 1]. [file 1471-2474-9-107-S2.doc]

| **Study** | **n / S** | **Age** | **Instr.** | **Pathology** | **Results** |  | **Defect detail** |
| --- | --- | --- | --- | --- | --- | --- | --- |
| Feeney et al. 2003 [25] | S=33 | 71 | Cadaver | RC tear (30%) | Deg. Hum./Glen. | 100% | Hum.: ant-inf  & post-sup  Glen.: ant-inf  & ant-sup |
|  |  | (49-93) |  | No RC tear (70%) | Deg. Hum./Glen. | 57%^1^ |  |
|  |  |  |  | Deg. Hum./Glen. (70%) | RC tear | 43% |  |
|  |  |  |  | No deg. Hum./Glen. (30%) | RC tear | 0% |  |
| Gartsman and Taverna 1997 [26] | n=200 | 56  (31-81) | AS | RC tear (100%) | Deg. Hum.  Deg. Glen. | 7%  7% | up to 2.5×2.5 cm^2^  up to 1.6×1.6 cm^2^ |
| Hsu et al. 2003 [1] | S=44 | 73  (62-86) | Cadaver | RC tear (32%) | Deg. Hum.  Deg. Glen. | 100%  100% | post; Area 36%  ant-inf; Area 32% |
|  |  |  |  | No RC tear (68%) | Deg. Hum.  Deg. Glen. | 100%  100% | Area 7%^2^  Area 6%^2^ |
| Konno et al. 2002 [30] | S=86 | 77  (54-100) | Cadaver | RC tear (35%) | Deg. Glen. | See^3^ | inf |
| Miller and Savoie 1994 [31] | n=100 | 55  (18-92) | AS | RC tear (100%) | Deg. Hum./Glen.  Passive dislocation | 28%  54% |  |
| Petersson 1983 [12] | S=151 | 68  (18-92) | Cadaver | Deg. Hum./Glen. (23%)  No deg. Hum./Glen. (77%) | RC deg. or tear | 76% |  |
|  |  |  |  |  | RC deg. or tear | 19%^4^ |  |
