# Supplementary material for: Post-traumatic glenohumeral cartilage lesions: a systematic review
Source: BMC Musculoskelet Disord. 2008 Jul 23;9:107. doi: 10.1186/1471-2474-9-107 (PMC2503981; doi:10.1186/1471-2474-9-107)
Supplement: Additional File 3 — Chronic traumas leading to GH cartilage lesions due to instability. The first two articles discuss the incidence of GH degeneration in unstable shoulders. The remaining papers report on single cases where all patients show signs of humeral and/or glenoidal degeneration. 1Numerous dislocations; 2No history of dislocation but pain at throwing; 3Numerous subluxations post; 4Luxation ant-inf; 5Numerous dislocations ant. For abbreviations, see [Additional file 1]. [file 1471-2474-9-107-S3.doc]

| **Study** | **n** | **Age** | **Instr.** | **Pathology** | **Associated findings** | **Results** | **Defect detail** | **Activity** |
| --- | --- | --- | --- | --- | --- | --- | --- | --- |
| Cameron et al. 2003 [22] | 422 | 40  (4-74) | AS | Instab.   - ant 76% - post 9% - multidir. 15% |  | Deg. Hum. 25% |  |  |
| Werner et al. 2003 [35] | 28 | 27 | AS | Instab. ant & ant-inf | Labral damage | Deg. Glen. 57% | ant | See^1^ |
| Romeo et al. 2002 [33] | 1 | 14 | AS | Instab. ant-inf | - GH joint capsule absent - Labral tear post-sup | Deg. Hum. | ant-sup  3.3×1.5 cm^2^ | Baseball^2^ |
| Siebold et al. 2003 [4] | 2 | 24  (16-31) | AS | Instab. post | Hyperlax. | Deg. Hum. | cent  2×2 cm^2^ | Swimming^3^ |
|  |  |  |  | Instab. ant | - Labral lesion - Hyperlax. | Deg. Hum. | 1.5×1.5 cm^2^ | Soccer^4^ |
| Scheibel et al. 2004 [34] | 6 | 44  (31-57) | AS | Hyperlax. (n=2) |  | Deg. Hum. | cent  up to 1.2×1.2 cm^2^ |  |
|  |  |  |  | Instab. (n=4) |  | Deg. Hum. (n=3)  Deg. Glen. (n=1) | post-cent  up to 1.4×1.4 cm^2^  ant-cent  1.1×1.1 cm^2^ |  |
| Yu et al. 1998 [36] | 1 | 33 | MRI | Instab. ant | Labral tear ant | Deg. Glen. | sup | Hockey^5^ |
