# Supplementary material for: Post-traumatic glenohumeral cartilage lesions: a systematic review
Source: BMC Musculoskelet Disord. 2008 Jul 23;9:107. doi: 10.1186/1471-2474-9-107 (PMC2503981; doi:10.1186/1471-2474-9-107)
Supplement: Additional File 4 — Chronic traumas leading to GH cartilage lesions due to impingement. 1Mainly sportive active men, e.g. weightlifter, tennis player; 2Near supraspinatus insertion. For abbreviations, see [Additional file 1]. [file 1471-2474-9-107-S4.doc]

| **Study** | **n** | **Age** | **Instr.** | **Pathology** | **Associated findings** | **Results** |  | **Defect detail** | **Activity** |
| --- | --- | --- | --- | --- | --- | --- | --- | --- | --- |
| Ellmann et al. 1992 [24] | 18 | 51  (21-67) | AS | Imping. |  | Deg. Hum.  Deg. Glen. | 100%  89% | mainly cent  mean 2.2×2.2 cm^2^  mainly ant-inf | Active men^1^ |
| Guntern et al. 2003 [27] | 52 | 46  (17-73) | AS | Clinical sub-  acromial imping. | - RC tear - Labral lesion | Deg. Hum.  Deg. Glen. | 29%  15% |  |  |
| Kaplan et al. 2004 [29] | 9 | no info | AS | Internal imping. | Labral abnormality | Deg. Hum.  Deg. Glen. | 11%  11% | Kissing lesion in ABER position | Throwing athletes |
| Paley et al. 2000 [32] | 41 | 25  (18-36) | AS | Internal imping. | RC fraying | Deg. Hum. | 17% | sup^2^ | Throwing athletes |
